# Natural disturbance reduces disease risk in endangered rainforest frog populations

**DOI:** 10.1038/srep13472

**Published:** 2015-08-21

**Authors:** Elizabeth A. Roznik, Sarah J. Sapsford, David A. Pike, Lin Schwarzkopf, Ross A. Alford

**Affiliations:** 1 College of Marine and Environmental Sciences, James Cook University, Townsville, Queensland 4811, Australia

## Abstract

Natural disturbances can drive disease dynamics in animal populations by altering the microclimates experienced by hosts and their pathogens. Many pathogens are highly sensitive to temperature and moisture, and therefore small changes in habitat structure can alter the microclimate in ways that increase or decrease infection prevalence and intensity in host populations. Here we show that a reduction of rainforest canopy cover caused by a severe tropical cyclone decreased the risk of endangered rainforest frogs (*Litoria rheocola*) becoming infected by a fungal pathogen (*Batrachochytrium dendrobatidis*). Reductions in canopy cover increased the temperatures and rates of evaporative water loss in frog microhabitats, which reduced *B. dendrobatidis* infection risk in frogs by an average of 11–28% in cyclone-damaged areas, relative to unaffected areas. Natural disturbances to the rainforest canopy can therefore provide an immediate benefit to frogs by altering the microclimate in ways that reduce infection risk. This could increase host survival and reduce the probability of epidemic disease outbreaks. For amphibian populations under immediate threat from this pathogen, targeted manipulation of canopy cover could increase the availability of warmer, drier microclimates and therefore tip the balance from host extinction to coexistence.

Ecosystems are heterogeneous and vary across space and time at multiple scales, reflecting responses to environmental changes and the histories of natural disturbances, such as wildfire, windstorms, and flooding^1^,^2^,^3^,^4^. Natural disturbances can alter ecosystem, community, and population structure, and they can modify resource availability and the physical environment^2^. Varying in size, frequency, and intensity, natural disturbances are important mechanisms for energy flow, nutrient cycling, and maintenance of biological and structural diversity in ecosystems^2^,^4^. For example, hurricanes play a fundamental role in maintaining species diversity in many tropical forests, and wildfires often maintain grassland ecosystems^4^. Within ecological communities, species may be positively or negatively affected by disturbances, which can lead to shifts in the interactions between species, such as competition, predation, and parasitism^1^,^5^.

Natural disturbances can strongly influence disease dynamics, but the nature of these effects depends on the ecology, physiology, and behaviour of both hosts and pathogens. Disturbances can directly reduce pathogen abundance in the environment, which can lead to lower transmission rates^6^. Alternatively, transmission rates can increase when habitat disturbances cause hosts to aggregate at high densities^7^,^8^. Complex disturbance-induced changes in the abundance and diversity of vectors, intermediate hosts, and/or final hosts can also influence transmission^9^. Disturbances can cause stress or deterioration in host body condition^10^,^11^, which can compromise their immune responses and increase susceptibility to disease^12^. Disturbances can also lead to changes in microclimatic conditions, which can influence host susceptibility by affecting their immune responses^13^,^14^ or their exposure to pathogens through changes in behaviour^15^,^16^,^17^,^18^. Because many pathogens are highly sensitive to temperature and moisture, small changes in these conditions caused by habitat disturbances can have important implications for their growth and survival, in hosts or in environmental reservoirs^19^,^20^,^21^,^22^.

The widespread amphibian disease chytridiomycosis, caused by the chytrid fungus *Batrachochytrium dendrobatidis*, is highly sensitive to the thermal and hydric environment (15–25 °C is optimal for growth, >28 °C is lethal, and it cannot tolerate desiccation^21^,^22^,^23^,^24^). Impacts of chytridiomycosis on amphibians are therefore often strongest in times and locations in which the environment is cool and moist, such as in the tropics during winter months and at higher elevations^25^,^26^,^27^. Impacts also differ within and among species because individual frogs can reduce their infection risk by selecting warmer and/or drier microenvironments^22^,^28^,^29^. Habitat composition also plays a role in mediating the interactions between amphibians and *B. dendrobatidis*^30^,^31^,^32^,^33^,^34^,^35^,^36^. Canopy cover is a primary driver of microclimate in forested areas, particularly tropical rainforests, because large trees slow air movement and regulate the amount of solar radiation reaching the forest floor^37^. Infection prevalence and intensity are therefore often lower in areas with lower vegetation density^30^,^31^,^32^,^33^,^34^,^35^,^36^, presumably because individuals experience warmer, drier conditions that are less favourable for pathogen growth and survival. Forests with low canopy cover may be important to amphibians at risk of infection by *B. dendrobatidis*, but many amphibian populations occur in areas with high levels of canopy cover that provide cool, moist conditions conducive to infection by *B. dendrobatidis*.

Structural damage can alter the microclimates available to animals living beneath the canopy^38^,^39^, and such changes could be important drivers of *B. dendrobatidis* infection dynamics because many forested areas are subject to natural disturbances that reduce canopy cover. Strong winds, such as those accompanying cyclones and hurricanes, can cause great damage to the canopy structure in tropical rainforests^38^,^39^. Understanding how these forces influence habitat structure, microclimatic conditions, and host-pathogen interactions is extremely difficult due to their stochastic nature in space and time. Our landscape-scale study of seasonal *B. dendrobatidis* infection dynamics in the endangered rainforest frog *Litoria rheocola* was unexpectedly impacted by Severe Tropical Cyclone Yasi in February 2011 (Fig. 1). Although all of the six study sites were in the path of the cyclone, some sites experienced no change in canopy cover, whereas in other sites, the canopy was substantially reduced. We predicted that these changes in canopy structure and microclimate caused by the cyclone would benefit frogs by reducing infection risk, and that these effects would be an immediate and positive outcome of storm systems that otherwise cause widespread destruction.

## Methods

### Ethics statement

All methods involving animals were carried out in accordance with the approved guidelines and protocols under permits A1420 and A1673 issued by the Animal Ethics Committee at James Cook University. The Queensland Department of Environment and Resource Management also approved all protocols involving animals and provided access to all field locations under permits WISP03070208 and WITK03070508.

### Study species

The common mistfrog (*Litoria rheocola*) is an IUCN Endangered species^40^ that occurs near fast-flowing rainforest streams in northeastern Queensland, Australia^41^. Males typically perch on rocks or streamside vegetation at night, and shelter among rocks or leaf litter in the streambed during the day^18^,^41^. Females are observed infrequently and likely spend more time away from streams than males^18^,^27^. This tropical species calls and breeds year-round, although reproductive behaviour decreases during the coolest weather^41^,^42^. Eggs are deposited in gelatinous masses beneath rocks in fast-flowing water, tadpoles hatch and feed on algae growing on rocks in riffles, and adults feed on a wide range of invertebrates^41^. By the mid-1990s, chytridiomycosis had extirpated *L. rheocola* from higher elevations (>400 m ASL) throughout its geographic range^43^. However, many populations have subsequently recovered or recolonized these areas^44^ and now coexist with the pathogen^27^,^45^. The prevalence and intensity of infection in *L. rheocola* are typically highest during cooler months, at higher elevations, and in areas where streams originate from high elevations^27^. Many individuals in this species carry sublethal *B. dendrobatidis* infections, sometimes for extended periods of time, and can ultimately recover^45^. Sublethal infections affect male calling effort in *L. rheocola*^42^, and may also affect other aspects of their behaviour, including patterns of movement, microhabitat use, and microenvironment use^29^.

### Study sites and cyclone path

**Figure 1:**
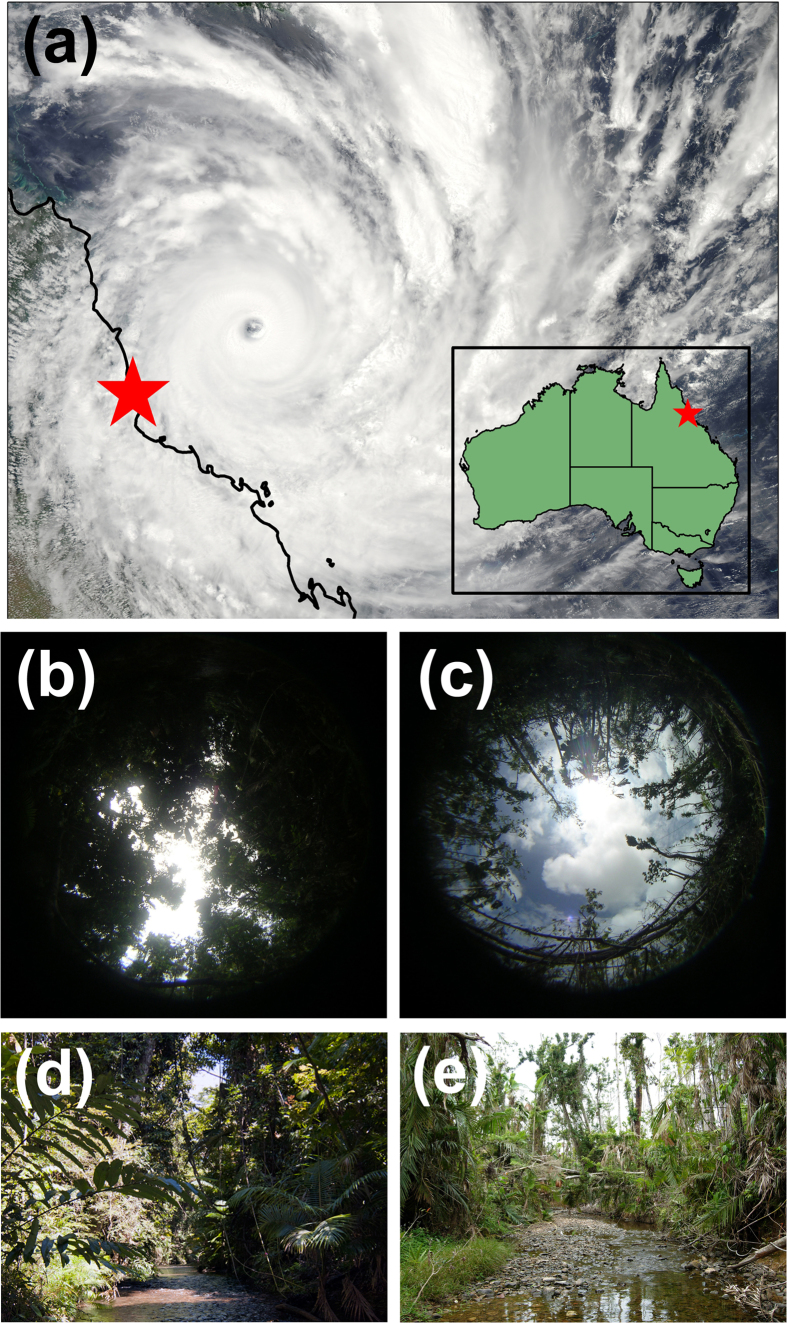
Severe Tropical Cyclone Yasi impacted the northeastern coast of Queensland, Australia, on 2–3 February 2011. Shown are (**a**) a satellite image of the cyclone approaching the coast (a star denotes our study region, and the inset shows this location within Australia), hemispherical photographs of the rainforest canopy above Stoney Creek taken from the same location at 80 m along our stream transect both (**b**) before and (**c**) after the cyclone and showing the canopy cover at that site (88% and 60%, respectively), and ground-level images of Stoney Creek (taken from different locations) both (**d**) before and (**e**) after the cyclone. Images were provided by (**a**) NASA (by the MODIS instrument on NASA’s Aqua satellite, taken at 13:35 Australian Eastern Standard Time on 2 February 2011. This image is not copyrighted and is used under NASA’s open access policy; the image is available at http://www.nasa.gov/images/content/514455main_Yasi-MODIS-WEDNESDAY-LARGE.jpg. We imported the image into ArcGIS 9.3 to create a map), (**b,c**) Sarah Sapsford, (**d**) Angus McNab, and (**e**) Elizabeth Roznik.

We studied disease dynamics at six rainforest streams in northeastern Queensland, Australia (Table 1; Fig. 1). Stream width varied from 5–10 m and streambeds were composed of rocks, ranging in size from small pebbles to large boulders (10 m in diameter). All streams contained pools, runs, and riffles, and most had several waterfalls. Our study began in June 2010; we sampled all six sites during winter (June-July) and spring (October-November) in 2010 (sampling methods described in detail below). On 2–3 February 2011, Severe Tropical Cyclone Yasi directly impacted our sites (Australian Category 5, Beaufort Scale 12; Fig. 1), bringing wind gusts up 285 kph, 5-m tidal storm surges, and up to 300 mm of rain over 24 hr^46^. The eye of the cyclone passed directly over two of our study sites (near the towns of Mission Beach and Tully^46^). Prior to the cyclone, streams were surrounded by tropical rainforest characterized by dense vegetation, including large trees (>10 m in height), vines, epiphytes, shrubs, and herbaceous plants. After the cyclone, we observed severe damage from the cyclone at some of our sites, with many trees uprooted or snapped off, and branches severely damaged and defoliated (Fig. 1). We quantified cyclone damage in March-April 2011 and sampled frogs during winter (June-July) and spring (October-November) in 2011.

### Forest canopy cover

To quantify effects of Cyclone Yasi on rainforest canopy cover, we compared hemispherical photographs of the canopy taken before (October-November 2010) and after (March-April 2011) the cyclone. We took photographs from the centre of the stream at 10-m intervals along a 400-m transect at each of our six study sites, and quantified the percentage of canopy cover using Gap Light Analyzer software^47^. To determine whether canopy cover was reduced significantly on average across sites, we compared the measurements at all locations before and after the cyclone using a one-tailed paired-difference t-test. We categorized a site as damaged when the canopy cover after the cyclone was significantly lower than it was before the cyclone. We took additional hemispherical photographs of the canopy in June-July 2011 (winter, 4–5 months post cyclone) and October 2011 (spring, 8 months post cyclone) during seasonal frog sampling. Measurements from the photographs closest to each sampling date at each site were included in the data used in our modelling of infection probability (described below) to account for any changes in local canopy cover that may have occurred between samples.

### Microenvironmental conditions

We determined whether variation in rainforest canopy cover influenced the microenvironmental conditions available to frogs by using physical models that mimic the thermal and hydric properties of frogs^48^,^49^. Each frog-shaped model was made of three percent agar and contained an embedded Thermochron iButton temperature datalogger (Maxim Integrated Products, California, USA; factory-calibrated and accurate to ± 0.5 °C) that was waterproofed to prevent failure from moisture damage^50^. These models lose and gain water at rates similar to frogs, and temperatures obtained from these permeable models are closely correlated with *L. rheocola* body temperatures^49^.

**Figure 2:**
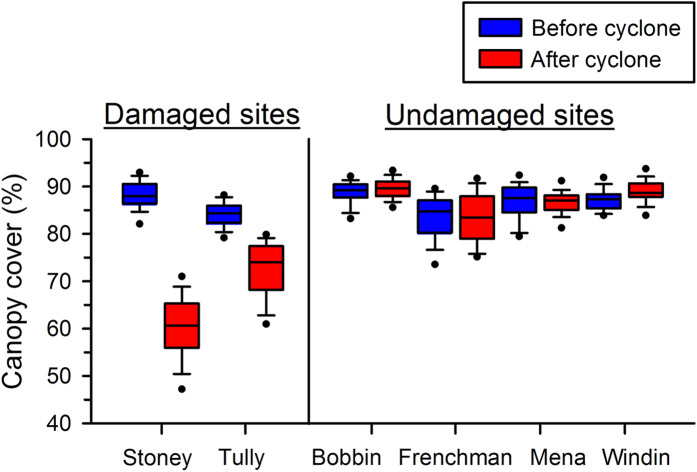
Boxplots of canopy cover (%) before and after Cyclone Yasi at two sites that were damaged significantly by the cyclone, and at four sites that were not damaged significantly (see Table 1 for statistical results).

We quantified the thermal and hydric conditions available to frogs under different levels of canopy cover by placing models on top of rocks in the streambed that are similar to rocks used by *L. rheocola*^18^. We placed 100 models on rocks along a 400-m section of stream at Frenchman Creek (a site with substantial variation in canopy cover; Fig. 2) for 24 hr in October 2011. We took a hemispherical photograph above each model, and determined canopy cover (%) using Gap Light Analyzer software^47^. Dataloggers recorded temperatures at 15-min intervals, which we used to calculate the mean temperature during the warmest part of the day (10:00–16:00) for each model. We also measured desiccation rates for model locations, expressed as the percentage of model mass lost due to water loss over 24 hr, by weighing each model (to 0.1 g) before and after field placement^48^,^51^. We used linear regressions to test for relationships between canopy cover and mean daytime temperature, and canopy cover and desiccation rate.

### Frog infection probability

We sampled adult male *L. rheocola* over five nights (one night in spring 2011) at each site during the winter (June-July) and spring (October-November) over a two-year period (2010–2011) that included samples before and after Cyclone Yasi. In *L. rheocola*, the prevalence of *B. dendrobatidis* is highest during these cooler months of the year^27^. We visually surveyed for frogs along 400-m transects marked at 10-m intervals using flagging tape. To determine whether frogs were infected by *B. dendrobatidis*, we swabbed the ventral surface and all four feet of each frog with a sterile rayon swab, covering these areas twice. These samples were analysed using real-time quantitative PCR assays^52^. Samples were run in triplicate and considered positive if at least two of the three PCR reactions were positive. We also gave each frog a unique identifying mark using visible implant elastomer^53^, ensuring that our sample of frogs was independent. For analysis, we used the initial capture of each frog (excluding recaptures, which were few), and used data only on males (determined by the presence of distinct nuptial pads; females and juveniles were few).

We used generalized linear mixed-effects models to examine the potential effects of canopy cover (arcsine-square root transformed percentage, expressed in degrees, using the nearest measurement in space and time along our stream transect for each frog capture), season (winter or spring) and year (2010 or 2011) on the probability of infection of individual frogs. Before Cyclone Yasi, we quantified canopy cover at all sites in October-November 2010, and after the cyclone, we quantified canopy cover at each site each time we sampled frogs. Infection status was coded as a binomial response variable, so we used models with a binomial family and a logit link function. We developed a set of candidate models that included models with all combinations of one, two, or three fixed effects, and all possible interactions. All models also included the random effect of site to control for any effects specific to particular sites. We ranked models according to Akaike’s Information Criterion with adjustment for finite sample size (AICc) to determine the strength of evidence for each model relative to the set of candidate models, using the criteria of Burnham and Anderson^54^. The models best supported by our data were averaged to produce a final model. These analyses were performed in program R, version 2.15.2^55^ using the lme4^56^ and MuMIn^57^ packages. Because infection loads were low during our study (98% of infected frogs had <50 zoospore equivalents; range: 1-913 zoospore equivalents), we could not examine possible effects of canopy cover on infection load.

## Results

Cyclone Yasi impacted a large area of the northeastern coast of Queensland, Australia (Fig. 1), but damage to rainforest canopy cover within this area was spatially heterogeneous. All six of our study sites were in the path of the cyclone, but only two underwent significant reductions in canopy cover; there was very little or no change in canopy cover at the other four sites (Table 1; Fig. 2). The eye of the cyclone passed directly over those two sites, but the degree of change in the canopy structure differed between them: Stoney Creek decreased much more dramatically (28% average reduction) than did Tully Creek (11% average reduction; Fig. 2). Canopy cover recovered only minimally during the eight-month period following the cyclone (4% average increase at Stoney Creek, and 3% average decrease at Tully Creek).

**Figure 3:**
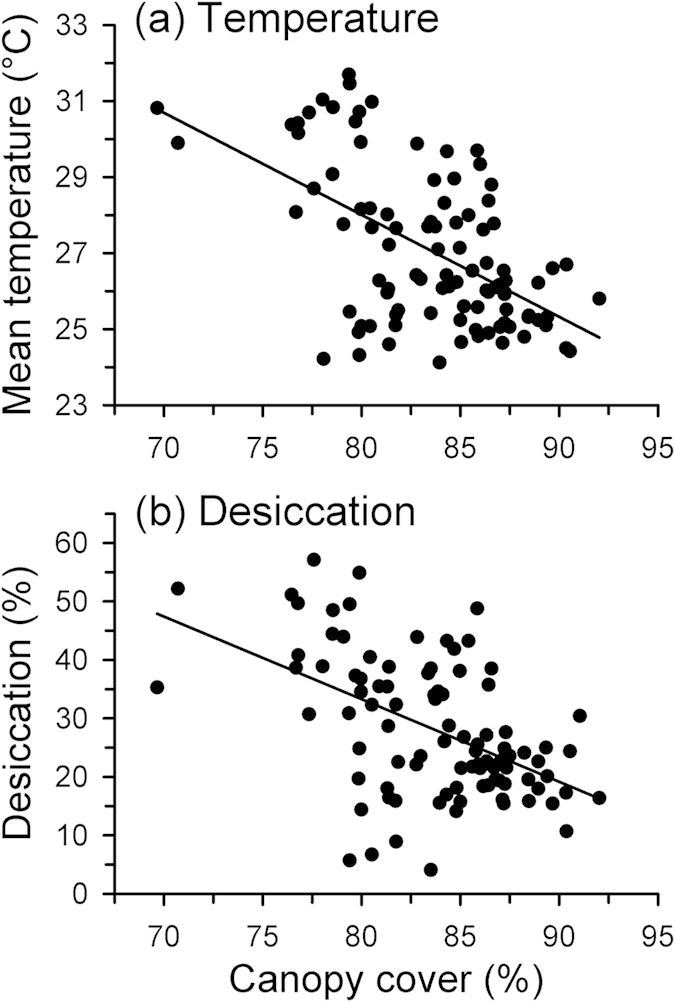
Relationships between canopy cover (%) and (a) mean temperature during the warmest part of the day (10:00–16:00), and (b) relative desiccation rate (percent of initial mass lost by models over 24 hr). These responses were estimated using physical models that mimic the thermal and hydric properties of frogs, which were placed on rocks in the stream that are similar to those used by *Litoria rheocola*.

Canopy cover significantly influenced the microclimatic conditions available to frogs on rocks in the streambed, in terms of both temperature and evaporative water loss, as estimated by data from our physical models (Fig. 3). Canopy cover was inversely related to both temperature (F_1,95_ = 41.874, R^2^ = 0.306, P < 0.001) and evaporative water loss (F_1,93_ = 41.874, R^2^ = 0.256, P < 0.001), indicating that increased canopy cover lowered temperature and increased moisture retention in frog microhabitats (Fig. 3).

**Figure 4:**
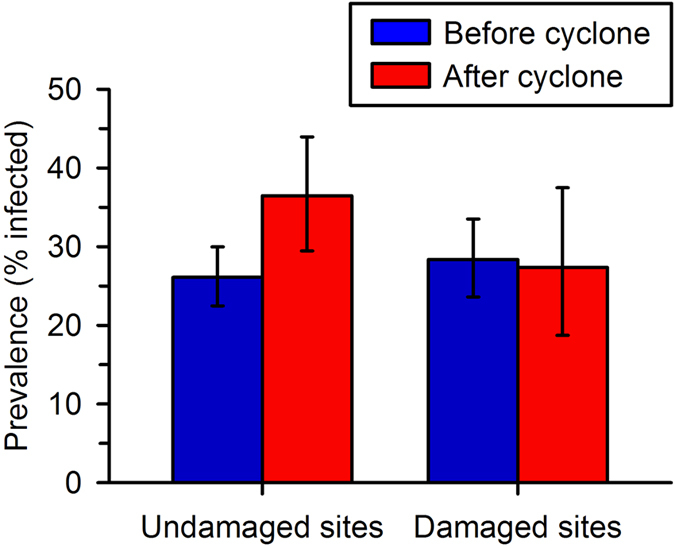
Prevalence of infection (and 95% confidence interval) by *Batrachochytrium dendrobatidis* in *Litoria rheocola* at sites before and after Cyclone Yasi that were or were not damaged significantly by the cyclone (see Table 1 for statistical results). Seasons and sites were combined to show the overall effect of the storm on infection prevalence.

We captured a total of 1163 unique male *L. rheocola* during four seasonal surveys at each of our six sites (Table 1). We did not find any evidence that the density of frogs at damaged sites changed relative to that at undamaged sites. The average number of frogs captured per night at both undamaged and damaged sites was slightly higher after the cyclone, and the average captures per night at damaged sites were similar to those at undamaged sites at both time points (Table 1). Before the cyclone, we captured an average of 14 and 17 frogs per night at undamaged and damaged sites, respectively. After the cyclone, we captured an average of 23 and 24 frogs per night at undamaged and damaged sites, respectively. At sites that were not damaged by the cyclone, the overall infection prevalence was 26.1% before the cyclone and 36.5% after the cyclone, and therefore higher during the second year of the study (Fig. 4). The infection prevalence at damaged sites was 28.4% before the cyclone and 27.4% after the cyclone; the initial prevalence was therefore similar to that of the undamaged sites, but prevalence after the cyclone was lower than that of the undamaged sites (Fig. 4).

**Figure 5:**
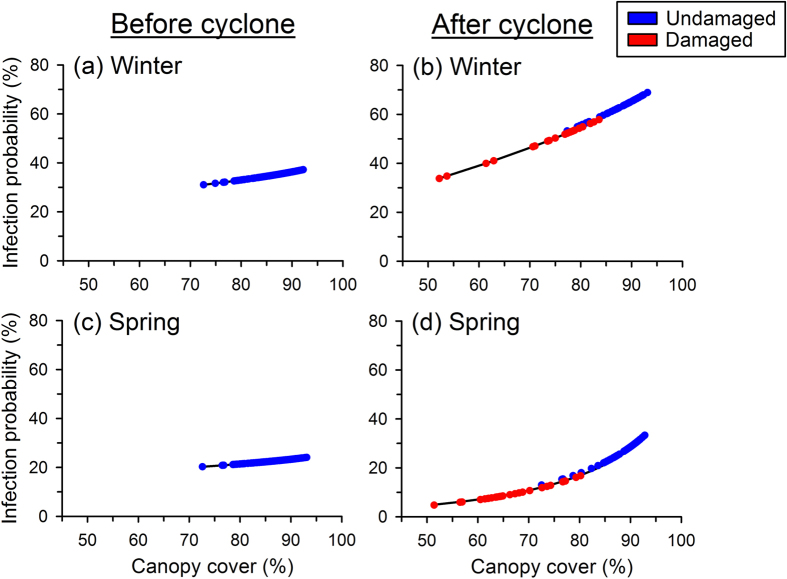
Probability of infection by the pathogen *Batrachochytrium dendrobatidis* for each individual frog (*Litoria rheocola*) sampled in our study during the winter (a,b) and spring (c,d) before and after Cyclone Yasi (2010–2011), based on the canopy cover above each frog’s location. Before the cyclone, all sites had intact, undamaged canopies, but after the cyclone, some sites had significantly damaged canopies. These predictions were generated from the averaged generalized linear mixed-effects model based on our field data (Table 2).

Our modelling exercise shows that canopy cover (%), year (2010 or 2011), and season (winter or spring) all influenced the infection probability of individual frogs (Table 2; Fig. 5). Five models were strongly supported by our data, each of which had similar ΔAICc values that were <3. Because the selected threshold for model selection should be based on all models in the set, rather than an arbitrary cutoff^58^, we included the top five models that were most strongly supported by our data and had a total Akaike weight of 98% (Table 2). We averaged these top five models to create a final model, which includes the random effect of site, the main effects of canopy cover, year, and season, and the interactions of canopy cover × year, season × year, year × season, and canopy cover × year × season (Table 2). Overall, frogs were more likely to be infected during winter than in spring, and infection probability was higher during the second year than in the first year (Fig. 5). Infection probability increased with canopy cover, and this relationship was stronger after the cyclone, when a much greater range in canopy cover was available at our sites overall (Fig. 5).

## Discussion

Cyclone Yasi had a positive effect on stream-breeding rainforest frogs (*Litoria rheocola*) living in cyclone-damaged areas by creating warmer and drier microhabitats that reduced the risk of infection by the chytrid fungus *Batrachochytrium dendrobatidis*. During the winter following the cyclone, the infection probability for frogs at cyclone-damaged sites ranged from 34–58%, whereas infection probabilities at undamaged sites were much higher, ranging from 52–68% (Fig. 5). This effect is likely due to increases in body temperatures and rates of evaporative water loss in hosts, both of which limit reproduction and survival of the pathogen^21^,^22^,^23^,^24^, as well as effects of temperature on the host’s defenses^13^,^14^,^59^. Other studies demonstrate that canopy structure can mediate the interactions between amphibians and *B. dendrobatidis* in a wide range of ecosystems in temperate and tropical regions. Stream-breeding amphibians are less susceptible to infection in deforested areas than in natural forest habitats^30^,^32^, and the impacts of chytridiomycosis are often lower in forest types with naturally sparse canopies^33^. For pond-breeding amphibians, infection risk is lower in habitats with vegetation densities that are naturally low or have been reduced by anthropogenic disturbance or wildfire^31^,^34^,^35^,^36^. Overall, canopy cover influences host-pathogen interactions in a wide range of ecosystems, and reductions in canopy cover caused by natural disturbances can provide an immediate benefit to frogs by decreasing their risk of infection.

In our frog populations, season and year significantly influenced the probability of infection, likely due to the effects of weather (Table 2). Infection risk was higher for frogs in winter than in spring because of cooler winter temperatures^27^. The weather also differed between the two years of our study: the mean minimum temperature during the months of sampling was lower in 2011 than 2010 (by 3.6 °C in winter, and 0.6 °C in spring), and the percentage of days above 25 °C was also lower in 2011 (by 31% in winter, and 2% in spring^60^). Both the observed infection prevalence and the predicted infection probability at sites with undamaged canopies were substantially higher during the second year of our study (Figs 4 and 5), likely because the cooler weather was conducive to faster pathogen growth rates^21^,^24^. However, the influence of cooler weather was quite distinct from the influence of canopy cover. Even in a year with relatively high infection risk (2011), the probability of infection was lower at the two cyclone-damaged sites than at the four undamaged sites (Figs 4 and 5). This demonstrates how more open canopies buffered frogs from the cooler temperatures and higher infection risk that frogs faced in shadier areas.

The benefits of canopy openings in reducing infection risk vary by season, year, and location (e.g., latitude, elevation), and also among species, depending on their habitat preferences and physiological tolerances. Even closely related species occurring in the same locations can have very different thermal and hydric preferences and patterns of microhabitat use^17^,^22^,^28^,^29^,^61^. Changes in canopy cover should more strongly affect species that prefer exposed areas^62^ or species that show no preference based on canopy cover, whereas species that prefer more closed canopies could behaviourally avoid canopy openings or override their effects by seeking sheltered microhabitats or by spending more time in water. Our study species, *L. rheocola*, often uses sheltered diurnal microhabitats, such as rock crevices^18^. Despite this, the effects of canopy cover still played a major role in influencing infection risk for this species, and these effects did not appear to reduce frog density at our sites. Other species and life stages, especially those that are aquatic, could also benefit from canopy disturbance when more open canopies lead to warmer water temperatures^34^,^36^,^63^. However, some species or life stages may be highly sensitive to desiccation and warm temperatures, and therefore may be unable to persist in areas with low canopy cover caused by disturbances^64^.

Increasing our understanding of the influence of habitat structure on host-pathogen interactions may help to identify amphibian populations most at risk from chytridiomycosis, and to identify and locate refuges from the disease^33^,^65^. Our findings also clearly suggest management strategies for reducing the impact of chytridiomycosis on frog populations. Providing canopy openings for populations at risk could be achieved using small-scale removal of individual trees or large branches, targeting vegetation shading critical habitat, such as ponds or stream sections (as has been achieved in other studies of amphibians and reptiles^66^,^67^). This technique has been used at ponds to successfully increase water temperature and amphibian diversity, with little evidence that any amphibian species was negatively affected^67^. Even small canopy openings that provide access to warm temperatures for short periods (e.g., one hour per day) may allow populations to persist that would otherwise be extirpated^33^,^68^. Targeted canopy reduction could be especially beneficial for those species under such severe threat from disease that only small populations remain. This strategy could be used to increase the success rates of reintroduction efforts or for *in situ* management of amphibians on the brink of extinction.

## Additional Information

**How to cite this article**: Roznik, E. A. *et al.* Natural disturbance reduces disease risk in endangered rainforest frog populations. *Sci. Rep.* **5**, 13472; doi: 10.1038/srep13472 (2015).

## Figures and Tables

**Table 1:** Study site details and sample sizes of unique male *Litoria rheocola* (N = 1163) captured at six rainforest streams in northeastern Queensland, Australia, that were impacted by Cyclone Yasi.

Site	Coordinates	Elevation (mASL)			Frog sample sizes				
			Cyclone damage		Before cyclone		After cyclone		
			t (df)	P	Infected	Uninfected	Infected	Uninfected	Total
Bobbin Bobbin Creek	17.378°S, 145.775°E	700	−1.967 (39)	0.972	35	99	24	13	171
Frenchman Creek	17.307°S, 145.922°E	40	1.073 (37)	0.145	33	152	24	40	249
Mena Creek	17.649°S, 145.987°E	60	0.330 (40)	0.371	59	121	12	34	226
Stoney Creek	17.920°S, 146.069°E	20	25.654 (35)	<0.001	50	75	10	26	161
Tully Creek	17.773°S, 145.645°E	150	12.954 (35)	<0.001	45	165	16	43	269
Windin Creek	17.365°S, 145.717°E	750	−2.944 (35)	0.997	17	36	6	28	87

Frogs were captured during seasonal stream surveys (1 night for spring surveys post-cyclone, and 5 nights for all other surveys) before and after the cyclone, and tested for infection by the pathogenic chytrid fungus *Batrachochytrium dendrobatidis* (infected or uninfected). Also shown are statistical results from one-tailed paired-difference t-tests for whether rainforest canopy cover decreased at each site after the cyclone.

**Table 2:** Generalized linear mixed-effects models (family: binomial, link function: logit) used to examine effects of changes in canopy cover caused by Cyclone Yasi on the probability of infection by *Batrachochytrium dendrobatidis* in individual *Litoria rheocola*.

Candidate models				
Model effects	AICc	ΔAICc	Weight	Cumulative weight
Canopy, Season, Year, Canopy × Year, Season × Year	1332.101	0.000	0.312	0.312
Canopy, Season, Year, Canopy × Season, Canopy × Year, Season × Year, Canopy × Season × Year	1332.326	0.224	0.279	0.591
Canopy, Season, Year, Season × Year	1333.541	1.440	0.152	0.743
Canopy, Season, Year, Canopy × Season, Canopy × Year, Season × Year	1333.584	1.482	0.149	0.892
Canopy, Season, Year, Canopy × Season, Season × Year	1334.625	2.524	0.088	0.980
Season, Year, Season × Year	1339.006	6.905	0.010	0.990
Canopy, Season, Year, Canopy × Season	1341.522	9.421	0.003	0.993
Canopy, Season, Year, Canopy × Season, Canopy × Year	1342.221	10.120	0.002	0.995
Canopy, Season, Year	1342.275	10.174	0.002	0.997
Canopy, Season, Year, Canopy × Year	1342.333	10.232	0.002	0.999
Canopy, Season, Canopy × Season	1347.617	15.516	0.000	0.999
Canopy, Season	1348.597	16.495	0.000	0.999
Season, Year	1349.068	16.967	0.000	0.999
Season	1351.864	19.763	0.000	0.999
Canopy, Year	1379.147	47.046	0.000	0.999
Canopy, Year, Canopy × Year	1379.977	47.876	0.000	0.999
Year	1384.527	52.426	0.000	0.999
Canopy	1384.689	52.588	0.000	0.999
Intercept only	1387.078	54.977	0.000	0.999
Final model				
Model effect	Estimate		Importance	
Intercept	- 1.846		−	
Canopy	0.018		1.00	
Season (spring)	- 0.314		1.00	
Year (2011)	- 1.130		1.00	
Season (spring) × Year (2011)	- 3.291		1.00	
Canopy × Year (2011)	0.042		0.75	
Canopy × Season (spring)	- 0.009		0.53	
Canopy × Season (spring) × Year (2011)	0.116		0.28	

We developed a set of candidate models that included all possible combinations of the following fixed effects, plus their interactions: canopy cover (%) at each frog’s location, season (winter or spring), and year (2010 or 2011). All models also included the random effect of site. We ranked models according to Akaike’s Information Criterion with adjustment for finite sample size (AICc). All models that we tested are shown, and five models were strongly supported by our data (∆AICc < 3, total weight of 98%). We averaged these five models to obtain the final model, which is presented below the candidate models.

## References

[b1] Sousa W. P. The role of disturbance in natural communities. Annual Review of Ecology and Systematics 15, 353–391 (1984).

[b2] White P. S. & Pickett S. T. A. Natural disturbance and patch dynamics: an introduction. In: The Ecology of Natural Disturbance and Patch Dynamics (ed. Pickett S. T. A.) 3–13 (Academic Press, Orlando, 1985).

[b3] Parminter J. Natural disturbance ecology. In: Conservation Biology Principles for Forested Landscapes (eds. Voller J. & Harrison S.) 3–41 (UBC Press, Vancouver, 1998).

[b4] Turner M. G., Gardner R. H. & O’Neill R. V. Landscape Ecology in Theory and Practice: Pattern and Process. (Springer-Verlag, New York, 2001).

[b5] Karr J. R. & Freemark K. E. Disturbance and vertebrates: an integrative perspective. In: The Ecology of Natural Disturbance and Patch Dynamics (eds. Pickett S. T. A. & White P. S.) 152–168 (Academic Press, Orlando, 1985).

[b6] Bendall J. F. Effects of fire on birds and mammals. In: Fire and Ecosystems (eds. Kozlowski T. T. & Ahlgren C. E.) 73–138 (Academic Press, New York, 1974).

[b7] Arneberg P., Skorping A., Grenfell B. & Read A. F. Host densities as determinants of abundance in parasite communities. Proceedings of the Royal Society B 265, 1283–1289 (1998).

[b8] Mbora D. N. M. & McPeek M. A. Host density and human activities mediate increased parasite prevalence and richness in primates threatened by habitat loss and fragmentation. Journal of Animal Ecology 78, 210–218 (2009). 19120603 10.1111/j.1365-2656.2008.01481.x

[b9] Behie A. M., Kutz S. & Pavelka M. S. Cascading effects of climate change: do hurricane-damaged forests increase risk of exposure to parasites? Biotropica 46, 25–31 (2014).

[b10] Wilson K. *et al.* In: The Ecology of Wildlife Diseases (eds. Hudson P. J., Rizzoli A., Grenfell B. T., Heesterbeek H. & Dobson A. P.) 6–44 (Oxford University Press, New York, 2001).

[b11] Jokela J., Taskinen J., Mutikainen P. & Kopp K. Virulence of parasites in hosts under environmental stress: experiments with anoxia and starvation. Oikos 108, 156–164 (2005).

[b12] Carey C., Cohen N. & Rollins-Smith L. Amphibian declines: an immunological perspective. Developmental and Comparative Immunology 23, 459–472 (1999). 10512457 10.1016/s0145-305x(99)00028-2

[b13] Wright R. K. & Cooper. E. L. Temperature effects on ectotherm immune responses. Developmental and Comparative Immunology 5 (supplement 1), 117–122 (1981).

[b14] Zapata A. G., Varas A. & Torroba M. Seasonal variations in the immune system of lower vertebrates. Immunology Today 13, 142–147 (1992). 1580995 10.1016/0167-5699(92)90112-K

[b15] Dowell S. F. Seasonal variation in host susceptibility and cycles of certain infectious diseases. Emerging Infectious Diseases 7, 369–374 (2001). 11384511 10.3201/eid0703.010301 PMC2631809

[b16] Altizer S. *et al.* Seasonality and the dynamics of infectious diseases. Ecology Letters 9, 467–484 (2006). 16623732 10.1111/j.1461-0248.2005.00879.x

[b17] Rowley J. J. L. & Alford R. A. Behaviour of Australian rainforest stream frogs may affect the transmission of chytridiomycosis. Diseases of Aquatic Organisms 77, 1–9 (2007). 17933392 10.3354/dao01830

[b18] Roznik E. A. & Alford R. A. Seasonal ecology and behavior of an endangered rainforest frog (*Litoria rheocola*) threatened by disease. PLoS ONE 10, e0127851 (2015). 25993520 10.1371/journal.pone.0127851 PMC4437910

[b19] Harvell C. D. *et al.* Climate warming and disease risks for terrestrial and marine biota. Science 296, 2158–2162 (2002). 12077394 10.1126/science.1063699

[b20] Murray K. A., Skerratt L. F., Garland S., Kriticos D. & McCallum H. Whether the weather drives patterns of endemic amphibian chytridiomycosis: a pathogen proliferation approach. PLoS ONE 8, e61061 (2013). 23613783 10.1371/journal.pone.0061061 PMC3629077

[b21] Stevenson L. A. *et al.* Variation in thermal performance of a widespread pathogen, the amphibian chytrid fungus *Batrachochytrium dendrobatidis*. PLoS ONE 8, e73830 (2013). 24023908 10.1371/journal.pone.0073830 PMC3762749

[b22] Stevenson L. A., Roznik E. A., Alford R. A. & Pike D. A. Host-specific thermal profiles affect fitness of a widespread pathogen. Ecology and Evolution 4, 4053–4064 (2014). 25505533 10.1002/ece3.1271 PMC4242559

[b23] Johnson M. L., Berger L., Phillips L., Speare R. Fungicidal effects of chemical disinfectants, UV light, desiccation and heat on the amphibian chytrid *Batrachochytrium dendrobatidis*. Diseases of Aquatic Organisms 57, 255–260 (2003). 14960039 10.3354/dao057255

[b24] Piotrowski J. S., Annis S. L. & Longcore J. E. Physiology of *Batrachochytrium dendrobatidis*, a chytrid pathogen of amphibians. Mycologia 96, 9–15 (2004). 21148822

[b25] Woodhams D. C. & Alford R. A. Ecology of chytridiomycosis in rainforest stream frog assemblages of tropical Queensland. Conservation Biology 19, 1449–1459 (2005).

[b26] Kinney V. C., Heemeyer J. L., Pessier A. P. & Lannoo M. J. Seasonal pattern of *Batrachochytrium dendrobatidis* infection and mortality in *Lithobates areolatus*: affirmation of Vredenburg’s “10,000 Zoospore Rule”. PLoS ONE 6, e16708 (2011). 21423745 10.1371/journal.pone.0016708 PMC3053364

[b27] Sapsford S. J., Alford R. A. & Schwarzkopf L. Elevation, temperature, and aquatic connectivity all influence the infection dynamics of the amphibian chytrid fungus in adult frogs. PLoS ONE 8, e82425 (2013). 24324786 10.1371/journal.pone.0082425 PMC3853199

[b28] Rowley J. J. L. & Alford R. A. Hot bodies protect amphibians against chytrid infection in nature. Scientific Reports 3, 1515 (2013). 23519020 10.1038/srep01515 PMC3604863

[b29] Roznik E. A. Effects of individual behaviour on host-pathogen interactions: Australian rainforest frogs and the chytrid fungus Batrachochytrium dendrobatidis (PhD thesis, James Cook University, Townsville, 2013).

[b30] Van Sluys M. & Hero J.-M. How does chytrid infection vary among habitats? The case of *Litoria wilcoxii* (Anura, Hylidae) in SE Queensland, Australia. Ecohealth 6, 576–583 (2009). 20155300 10.1007/s10393-010-0278-1

[b31] Raffel T. R., Michel P. J., Sites E. W. & Rohr J. R. What drives chytrid infections in newt populations? Associations with substrate, temperature, and shade. Ecohealth 7, 526–536 (2010). 21125308 10.1007/s10393-010-0358-2

[b32] Becker C. G. & Zamudio K. R. Tropical amphibian populations experience higher disease risk in natural habitats. Proceedings of the National Academy of Sciences 108, 9893–9898 (2011). 10.1073/pnas.1014497108 PMC3116417 21628560

[b33] Puschendorf R. *et al.* Environmental refuge from disease-driven amphibian extinction. Conservation Biology 25, 956–964 (2011). 21902719 10.1111/j.1523-1739.2011.01728.x

[b34] Becker C. G., Rodriguez D., Longo A. V., Talaba A. L. & Zamudio K. R. Disease risk in temperate amphibian populations is higher at closed-canopy sites. PLoS ONE 7, e48205 (2012). 23118953 10.1371/journal.pone.0048205 PMC3485156

[b35] Hossack B. R., Lowe W. H., Ware J. L. & Corn P. S. Disease in a dynamic landscape: host behavior and wildfire reduce amphibian chytrid infection. Biological Conservation 157, 293–299 (2013).

[b36] Beyer S. E., Phillips C. A. & Schooley R. L. Canopy cover and drought influence the landscape epidemiology of an amphibian chytrid fungus. Ecosphere 6, 78 (2015).

[b37] Whitmore T. C. An Introduction to Tropical Rain Forests. 2nd edn (Oxford University Press, New York, 1998).

[b38] Turton S. M. & Siegenthaler D. T. Immediate impacts of a severe tropical cyclone on the microclimate of a rain-forest canopy in north-east Australia. Journal of Tropical Ecology 20, 583–586 (2004).

[b39] Pohlman C. L., Goosem M. & Turton S. M. Effects of Severe Tropical Cyclone Larry on rainforest vegetation and understorey microclimate near a road, powerline and stream. Austral Ecology 33, 503–515 (2008).

[b40] IUCN. *The IUCN Red List of Threatened Species*. Version 2014.3. (2014) Available at: http://www.iucnredlist.org. (Accessed: 5th March 2015).

[b41] Dennis A. J. Common mistfrog, *Litoria rheocola*. In: Queensland’s Threatened Animals (eds. Curtis L. K., Dennis A. J., McDonald K. R., Kyne P. M. & Debus S. J. S.) 166–167 (CSIRO Publishing, Collingwood, 2012).

[b42] Roznik E. A., Sapsford S. J., Pike D. A., Schwarzkopf L. & Alford R. A. Condition-dependent reproductive effort in frogs infected by a widespread pathogen. Proceedings of the Royal Society B 282, 20150694 (2015). 10.1098/rspb.2015.0694 PMC4590479 26063847

[b43] McDonald K. & Alford R. A review of declining frogs in northern Queensland. In: Declines and Disappearances of Australian Frogs (ed. Campbell A.) 14–22 (Environment Australia, Canberra, 1999).

[b44] McDonald K. R., Mendez D., Muller R., Freeman A. B. & Speare R. Decline in the prevalence of chytridiomycosis in frog populations in North Queensland, Australia. Pacific Conservation Biology 11, 114–120 (2005).

[b45] Sapsford S. J. Population and disease dynamics of the amphibian chytrid fungus in the stream-associated frog Litoria rheocola. (MSc thesis, James Cook University, Townsville, 2012).

[b46] Australian Bureau of Meteorology. *Severe Tropical Cyclone Yasi*. (2014) Available at: http://www.bom.gov.au/cyclone/history/yasi.shtml. (Accessed: 1st October 2014).

[b47] Frazer G. W., Canham C. D. & Lertzman K. P. Gap Light Analyzer (GLA), version 2.0: image processing software to analyze truecolor, hemispherical canopy photographs. Bulletin of the Ecological Society of America 81, 191–197 (2000).

[b48] Rowley J. J. L. & Alford R. A. Models in field studies of temperature and moisture. In: Amphibian Ecology and Conservation: A Handbook of Techniques (ed. Dodd C. K. Jr .) 387–406 (Oxford University Press, New York, 2010).

[b49] Roznik E. A. & Alford R. A. Using pairs of physiological models to estimate temporal variation in amphibian body temperature. Journal of Thermal Biology 45, 22–29 (2014). 25436947 10.1016/j.jtherbio.2014.07.005

[b50] Roznik E. A. & Alford R. A. Does waterproofing Thermochron iButton dataloggers influence temperature readings? Journal of Thermal Biology 37, 260–264 (2012).

[b51] Schwarzkopf L. & Alford R. A. Desiccation and shelter-site use in a tropical amphibian: comparing toads with physical models. Functional Ecology 10, 193–200 (1996).

[b52] Boyle D. G., Boyle D. B., Olsen V., Morgan J. A. T. & Hyatt A. D. Rapid quantitative detection of chytridiomycosis (*Batrachochytrium dendrobatidis*) in amphibian samples using real-time Taqman PCR assay. Diseases of Aquatic Organisms 60, 141–148 (2004). 15460858 10.3354/dao060141

[b53] Sapsford S. J., Roznik E. A., Alford R. A. & Schwarzkopf L. Visible implant elastomer marking does not affect short-term movements or survival rates of the treefrog *Litoria rheocola*. Herpetologica 70, 23–33 (2014).

[b54] Burnham K. P. & Anderson D. R. Model Selection and Multi-Model Inference: A Practical Information-Theoretic Approach. 2nd edn (Springer-Verlag, New York, 2002).

[b55] R. Core Team. *R: A language and environment for statistical computing*. Version 2.15.2. (2012) Available at: http://www.R-project.org. (Accessed: 1st October 2014).

[b56] Bates D., Maechler M. & Bolker B. *lme4: Linear mixed-effects models using S4 classes*. (2012) Available at: http://cran.r-project.org/web/packages/lme4/index.html. (Accessed: 1st October 2014).

[b57] Barton K. *MuMIn: Multi-model inference*. (2012) Available at: http://cran.r-project.org/web/packages/MuMIn/index.html. (Accessed: 1st October 2014).

[b58] Burnham K. P., Anderson D. R. & Huyvaert K. P. AIC model selection and multimodel inference in behavioral ecology: some background, observations, and comparisons. Behavioral Ecology and Sociobiology 65, 23–35 (2011).

[b59] Daskin J. H., Bell S. C., Schwarzkopf L. & Alford R. A. Cool temperatures reduce antifungal activity of symbiotic bacteria of threatened amphibians—implications for disease management and patterns of decline. PLoS ONE 9, e100378 (2014). 24941262 10.1371/journal.pone.0100378 PMC4062522

[b60] Australian Bureau of Meteorology. *Climate data online*. (2014) Available at: http://www.bom.gov.au/climate/data. (Accessed: 1st October 2014).

[b61] Rowley J. J. L. & Alford R. A. Movement patterns and habitat use of rainforest stream frogs in northern Queensland, Australia: implications for extinction vulnerability. Wildlife Research 34, 371–378. (2007).

[b62] Roznik E. A. & Johnson S. A. Canopy closure and emigration by juvenile gopher frogs. Journal of Wildlife Management 73, 260–268 (2009).

[b63] Forrest M. J. & Schlaepfer M. A. Nothing a hot bath won’t cure: infection rates of amphibian chytrid fungus correlate negatively with water temperature under natural field settings. PLoS ONE 6, e28444 (2011). 22205950 10.1371/journal.pone.0028444 PMC3244395

[b64] Rittenhouse T. A. G., Harper E. B., Rehard L. R. & Semlitsch R. D. The role of microhabitats in the desiccation and survival of anurans in recently harvested oak-hickory forest. Copeia 2008, 807–814 (2008).

[b65] Puschendorf R., Hodgson L., Alford R. A., Skerratt L. F. & VanDerWal J. Underestimated ranges and overlooked refuges from amphibian chytridiomycosis. Diversity and Distributions 19, 1313–1321 (2013).

[b66] Pike D. A., Webb J. K. & Shine R. Removing forest canopy restores a reptile assemblage. Ecological Applications 21, 274–280 (2011). 21516904 10.1890/09-2394.1

[b67] Skelly D. K., Bolden S. R. & Freidenburg L. K. Experimental canopy removal enhances diversity of vernal pond amphibians. Ecological Applications 24, 340–345 (2014). 24689145 10.1890/13-1042.1

[b68] Daskin J. H., Alford R. A. & Puschendorf R. Short-term exposure to warm microhabitats could explain amphibian persistence with *Batrachochytrium dendrobatidis*. PLoS ONE 6, e26215 (2011). 22028834 10.1371/journal.pone.0026215 PMC3196517

